# Effects of Louse Bites

**Published:** 1919-07-26

**Authors:** 


### Effects of Louse Bites.

In the Archives of Intern. Med., April 15, Drs. Hirschfleur and Moore published the results of their experiments undertaken to determine whether the previously recorded observations represents a peculiarity of the individual on whom lice had fed, or whether it might be regarded as a general phenomena.

### A New Experiment.

Four perfectly healthy young men volunteered for the experiments. Each of the subjects allowed himself to be bitten twice daily by the number of lice specified. These lice were raised from eggs and had never fed on any except healthy individuals. In every individual bitten, except one, there was a prompt rise of temperature, ranging from 99.3 to 99 F., after the lice had been fed. Sometimes this occurred with surprising rapidity, and the temperature reached 99.9 F. within an hour after feeding. In three out of the four cases a well-defined rash, composed of semilunar and crescentic maculæ from 2 to 3 mm. in size, resembling those of a fading measles or German measles, occurred. The rash was not very striking, and yet was definite enough to be seen without difficulty when it was at its height. The maculæ disappeared on pressure. It was distributed over the chest, back and upper abdomen, and in no case appeared on the face, neck, arms or lower limbs. It was always most distinct, and persisted longest in the regions between the nipples and the lower costal margins.

### Toxic Effect of Normal Lice.

The authors conclude that their observations point strongly toward the presence of a substance in the uninfected louse sufficiently toxic to give rise to a generalised skin eruption and mild fever. Apart from such considerations, which prove definite constitutional disturbance, it is quite obvious that men who are subject to louse bites have a lower mental and bodily vigour, and that, other things being equal, a louse-free army would be considerably better fighting men than the same army louse-infected.

For the latter facts at least not a few M.O.s recently with our fighting forces can give ample evidence, for it is not only in such simple toxic effects that the louse can exert its deadly influence upon armies. There is the news from the Near Eastern theatres of war, where these pests still continue to spread death-dealing disease far and wide. In fact, the deciding factor in these areas is said to be the louse, who even to the present day carries all before him and indirectly defeats, immediately or ultimately, almost every attempt at military reorganisation.

